# Towards Post-Genomic Oncology: Embracing Cancer Complexity via Artificial Intelligence, Multi-Targeted Therapeutics, Drug Repurposing, and Innovative Study Designs

**DOI:** 10.3390/ijms26167723

**Published:** 2025-08-10

**Authors:** Annabella Di Mauro, Massimiliano Berretta, Mariachiara Santorsola, Gerardo Ferrara, Carmine Picone, Giovanni Savarese, Alessandro Ottaiano

**Affiliations:** 1 Istituto Nazionale Tumori di Napoli, IRCCS “G. Pascale”, Via M. Semmola, 80131 Naples, Italy; annabella.dimauro@istitutotumori.na.it (A.D.M.); mariachiara.santorsola@istitutotumori.na.it (M.S.); gerardo.ferrara@istitutotumori.na.it (G.F.); c.picone@istitutotumori.na.it (C.P.); 2 Department of Clinical and Experimental Medicine, University of Messina, 98125 Messina, Italy; mberretta@unime.it; 3 Department of Genetics, AMES, Centro Polidiagnostico Strumentale srl, Via Padre Carmine Fico 24, 80013 Casalnuovo Di Napoli, Italy; giovanni.savarese@centroames.it

**Keywords:** target therapy, next-generation sequencing, precision oncology, artificial intelligence, tumor heterogeneity, drug repurposing

## Abstract

Recent advances in precision oncology have led to significant breakthroughs through the targeting of defined oncogenic drivers. However, the clinical efficacy of single-target therapies is increasingly constrained by the intrinsic complexity and adaptability of cancer. Solid tumors frequently arise from multifactorial oncogenic processes and adapt via diverse resistance mechanisms, ultimately limiting the durability of monotherapies. This review advocates for a paradigm shift toward multi-targeted, AI-enhanced strategies that harness high-throughput multi-omic data to inform the rational design of combination therapies. By leveraging artificial intelligence for drug discovery and repurposing, response prediction, and clinical trial optimization, the field of oncology is poised to transcend reductionist approaches and more fully address the biological intricacy of cancer.

## 1. Introduction

Cancer is a multifaceted and dynamic disease, shaped by the complex interplay among genomic alterations, signaling redundancy, clonal evolution, and the surrounding tumor microenvironment. In recent years, the paradigm of precision oncology—focused on targeting specific oncogenic drivers—has led to significant therapeutic progress. However, growing evidence underscores the limitations of single-target strategies in the context of tumor heterogeneity, resistance mechanisms, and pathway plasticity.

In response to this evolving complexity, the concept of “continuously responsive oncology” is particularly compelling. This paradigm envisions cancer treatment as a dynamic and iterative process, guided by real-time molecular data, adaptive therapeutic combinations, and predictive modeling, capable of responding to the evolving biology of each patient’s tumor. Instead of relying on fixed, one-time treatment decisions, this approach seeks to continuously incorporate new layers of information to refine and optimize therapeutic strategies over time.

This scoping review aims to map emerging conceptual and technological trajectories, including the integration of artificial intelligence (AI), that have the potential to redefine future cancer treatment paradigms. While not intended as a comprehensive overview, it offers a forward-looking perspective on how the limitations of monotherapy are being addressed through systems-level, multi-targeted, and computationally guided strategies.

The review is structured around several key dimensions of cancer complexity. First, it delineates the mechanistic limitations of single-target inhibition. It then examines the rationale for and outcomes of combination therapies, emphasizing their potential to counteract pathway compensation and clonal selection. The subsequent sections explore how liquid biopsy and next-generation sequencing (NGS) technologies enable real-time tracking of tumor evolution, and how machine learning models applied to these datasets can predict resistance trajectories and inform treatment selection. A dedicated section addresses the critical yet often underappreciated role of the tumor microenvironment (TME), highlighting advanced spatial and single-cell technologies that facilitate high-resolution profiling of TME dynamics. The final sections delve into synthetic lethality and drug repurposing, uncovering novel pharmacological opportunities that may be further accelerated through AI-driven discovery pipelines. By synthesizing key axes of cancer biology—including tumor-intrinsic complexity, evolutionary dynamics, immune contexture, and computational modeling—this review proposes a forward-facing framework for future oncology. It encourages readers to move beyond the linear “target-and-block” approach and embrace a more integrated, adaptive, and AI-enhanced vision for cancer care.

## 2. Limitations of the Single-Target Approach in the Precision Oncology Paradigm

In contemporary oncology, the paradigm of precision medicine has ushered in transformative therapeutic strategies, particularly through the identification and targeting of dominant oncogenic drivers. This approach has yielded significant clinical benefits in malignancies such as chronic myeloid leukemia (CML) with *BCR*-*ABL1* fusion and *NTRK* fusion-positive tumors, where targeted monotherapies have demonstrated remarkable efficacy [1,2]. Nonetheless, resistance inevitably emerges in a substantial subset of patients. In CML, secondary kinase domain mutations—most notably the p.T315I gatekeeper mutation—confer high-level resistance to first- and second-generation *BCR*-*ABL1* inhibitors, necessitating the development of third-generation agents such as ponatinib [3]. Similarly, in tumors harboring *NTRK* gene fusions, acquired resistance often arises through solvent front mutations (e.g., *NTRK1* p.G595R, *NTRK3* p.G623R) or xDFG motif substitutions that impair drug binding, prompting the design of next-generation inhibitors with improved conformational tolerance [4]. These examples illustrate how tumor plasticity and selective pressure drive the emergence of resistance, highlighting the importance of anticipatory therapeutic strategies to prolong clinical benefit. Even if not exhaustive, additional examples can be provided to illustrate the efficacy of biologically targeted monotherapies in tumors harboring defined molecular lesions. In non-small cell lung cancer (NSCLC), activating mutations in the epidermal growth factor receptor (*EGFR*) gene have been effectively targeted by tyrosine kinase inhibitors (TKIs) such as erlotinib, resulting in improved progression-free survival. However, resistance mechanisms, including secondary mutations, such as p.T790M and the activation of alternative signaling pathways, often emerge, necessitating the development of next-generation inhibitors [5]. Similarly, rearrangements involving the anaplastic lymphoma kinase (*ALK*) gene in NSCLC have been successfully targeted by crizotinib. Despite initial responses, resistance frequently develops through secondary *ALK* mutations, gene amplification, or activation of bypass signaling pathways, leading to the exploration of second-generation *ALK* inhibitors to overcome resistance [6]. In melanoma, the *BRAF* p.V600E mutation has been a critical target, with inhibitors such as vemurafenib demonstrating substantial clinical activity. Nevertheless, resistance mechanisms such as *NRAS* mutations, *BRAF* splice variants, and the activation of parallel pathways such as PI3K/AKT have been identified, prompting combination therapies to enhance efficacy [7]. *HER2* amplification in breast cancer has been effectively targeted by trastuzumab, improving survival rates. Yet, resistance can occur through mechanisms such as PI3K pathway activation, expression of truncated HER2 forms (p95HER2), and cross-talk with other receptors, leading to the development of additional HER2-targeted agents and combination therapies to address resistance [8].

**Figure 1:**
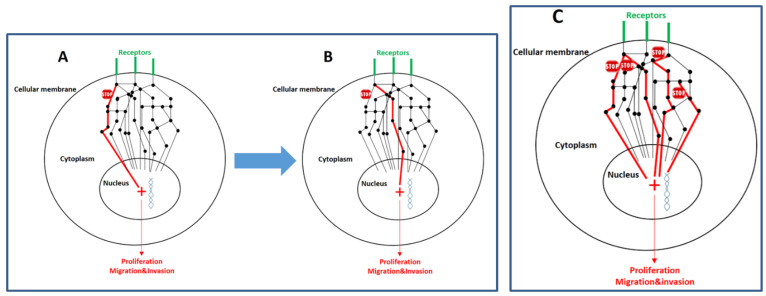
Cancer represents the phenotypic outcome of a multitude of mutations that disrupt intracellular signaling pathways. These molecular pathways are inherently redundant and highly interconnected. Several intracellular nodes (depicted as black spheres) function as hubs for signal communication, integration, regulation, amplification, or attenuation. The flow of these signals, ultimately transmitted to the nucleus, is represented by black lines. The mutated and hyperactivated signaling cascades are highlighted in red lines and depicted with increased line thickness. The stops in red represent potentially druggable pathways. It is within the nucleus that the cell modulates gene expression programs encoding proteins responsible for key cellular behaviors such as proliferation, migration, and invasion (three hallmark capabilities that are hyperactivated and deregulated in malignant cells). Panel (**A**) illustrates a simplified view of a signaling pathway downstream of three surface receptors. Due to the genetic and evolutionary plasticity of neoplastic cells, inhibition of a single pathway often results in the scenario depicted in Panel (**B**), where not only alternative signaling routes become activated, but also a selective advantage is conferred to subclones already capable of exploiting such compensatory mechanisms. As a consequence, the tumor mass evolves towards a composition enriched in cells that sustain proliferation and metastatic dissemination through these alternative pathways. However, the scenario illustrated in Panel (**C**) is more realistic: the malignant phenotype arises from multiple coexisting alterations that require comprehensive identification and simultaneous inhibition. In all cases, the volume and complexity of biological information to be processed is substantial (including signaling cascades, regulatory nodes, feedback mechanisms, the dynamic evolution of molecular alterations over time, etc.). For this reason, the integration of artificial intelligence with multi-targeted pathway inhibition represents a promising and necessary therapeutic frontier.

In other words, cancer is not a static entity, but rather a dynamic, evolving ecosystem shaped by intratumor heterogeneity, genomic instability, and the selective pressures exerted by the TME [9]. In many solid tumors, the presence of multiple co-occurring mutations reflects a non-linear and multifactorial oncogenic trajectory. In this context, targeting a single signaling node is often insufficient to achieve sustained clinical benefit. Resistance—whether intrinsic or acquired—frequently arises through pathway redundancy, compensatory signaling mechanisms, or the clonal selection of resistant subpopulations [10]. Figure 1 shows a schematic representation highlighting the complexity of intracellular signaling pathways and how this intricacy impacts their therapeutic inhibition.

## 3. Rationale for Combination Therapies Involving Biologic Agents

A concrete example illustrating the limitations of single-target approaches and the therapeutic potential of multi-target inhibition is the combination of sotorasib (a *KRAS* p.G12C inhibitor) and panitumumab (an anti-EGFR monoclonal antibody) in advanced and chemotherapy-refractory colorectal cancer. Dual blockade of both KRAS-dependent and EGFR-mediated signaling pathways led to significantly improved progression-free survival and objective response rates compared to standard therapies [11]. This underscores the clinical benefit of simultaneously targeting multiple oncogenic drivers.

Beyond this recent example, where the objective response rate (ORR) increased from 0% with sotorasib monotherapy to 26.4% when combined with panitumumab, there are additional compelling instances across oncology in which the strategic combination of targeted agents has resulted in a marked improvement in tumor response. Without claiming to be exhaustive, in HER2-positive breast cancer, the combination of trastuzumab and lapatinib significantly outperformed trastuzumab alone, achieving a pathologic complete response (pCR) rate of 51.3% versus 29.5% in the neoadjuvant setting [12]. Similarly, in the hormone receptor-positive/HER2-positive metastatic breast cancer population, the triplet regimen of trastuzumab, lapatinib, and an aromatase inhibitor yielded an ORR of 31.7%, clearly superior to the 13.7% observed with the dual combination of trastuzumab and endocrine therapy [13]. In *BRAF* V600-mutant melanoma, the addition of the MEK inhibitor cobimetinib to the BRAF inhibitor vemurafenib increased the ORR from 45% to 68% [14]. These studies converge on a unifying principle: cancer results from multiple dysregulated biological pathways, and rational combinations of targeted agents can produce therapeutic synergy and enhanced tumor regression (Table 1). In other words, simultaneous inhibition of several altered nodes—whether convergent within the same pathway or divergent across distinct pathways—is a rational strategy that may enhance therapeutic efficacy by comprehensively targeting tumor complexity and signaling redundancy.

## 4. Mapping Tumor Evolution Through Liquid Biopsy: A Road to Precision Oncology Empowered by Artificial Intelligence

The development of acquired resistance to therapy, which accounts for approximately 90% of cancer-related deaths, remains one of the foremost challenges in contemporary oncology. At the core of this issue lies the emergence of drug-tolerant persister (DTP) cells that survive initial therapeutic assaults and serve as reservoirs for clonal evolution, facilitating the development of genetically distinct subpopulations and fueling intratumoral heterogeneity (ITH) [15,16]. ITH, a hallmark of malignancy, is not a static phenomenon but a dynamic and adaptive process characterized by spatial and temporal variability in tumor molecular architecture. Traditionally, our understanding of ITH and resistance mechanisms has relied on serial tissue biopsies, which, although informative, present several critical limitations—including procedural invasiveness, sampling bias, and the inability to capture the full extent of tumor heterogeneity over time, particularly in metastatic or anatomically inaccessible lesions. In contrast, liquid biopsy, particularly the genomic analysis of circulating tumor DNA (ctDNA) and other circulating biomarkers, has emerged as the most advanced and clinically viable tool for capturing tumor dynamics in a minimally invasive and temporally resolved manner [17]. Liquid biopsy enables real-time monitoring of tumor evolution by facilitating the detection of emerging resistance-associated mutations, subclonal dynamics, and longitudinal assessment of molecular alterations in response to therapy. By providing a comprehensive snapshot of the genetic landscape across multiple tumor sites, it offers a holistic view of spatial heterogeneity and therapeutic adaptation. Importantly, liquid biopsy can detect low-frequency variants and clonal expansions that precede radiological progression, enabling earlier therapeutic interventions and adaptive treatment strategies [18,19].

Moreover, integration of NGS technologies with liquid biopsy has significantly expanded its utility. High-depth targeted panels, whole-exome sequencing (WES), and even emerging whole-genome approaches applied to ctDNA allow for the reconstruction of tumor phylogenies, identification of driver mutations, and mapping of resistance trajectories over time [20]. The convergence of liquid biopsy with computational oncology and machine learning algorithms further enables prediction of resistance pathways, inference of subclonal architecture, and development of patient-specific models of tumor progression [21]. However, widespread adoption of liquid biopsy and comprehensive genetic assessment in routine oncology practice is hindered by several barriers. Technologically, improvements in ctDNA detection sensitivity and specificity remain necessary—especially in early-stage disease or low-shedding tumors—as do standardization of pre-analytical variables, sequencing protocols, and bioinformatic pipelines to ensure reproducibility and clinical reliability [22]. Furthermore, economically, high-throughput sequencing remains costly, particularly in resource-limited healthcare systems [23]. Cost reduction strategies include the development of targeted panels focused on clinically actionable mutations, streamlined analytical workflows, and the incorporation of liquid biopsy into broader diagnostic and treatment algorithms to establish clear cost-effectiveness. Additionally, regulatory and reimbursement frameworks must evolve to support clinical utility beyond niche applications. Ethical and logistical challenges also accompany dynamic genomic surveillance: sampling frequency, interpretation of low-level variants, management of incidental findings, and psychological impact on patients require careful policy and clinical guidelines. Nonetheless, the trajectory of precision oncology is moving toward more agile, minimally invasive, and data-rich approaches, with liquid biopsy and real-time genetic profiling at the forefront.

As the clinical use of liquid biopsy expands, its role is evolving from a passive monitoring tool to an active predictor of therapeutic response and tumor evolution. During the course of treatment, serial genetic reassessments through ctDNA analysis can reveal the emergence of resistance-conferring mutations before clinical progression becomes evident [24].

**Figure 2:**
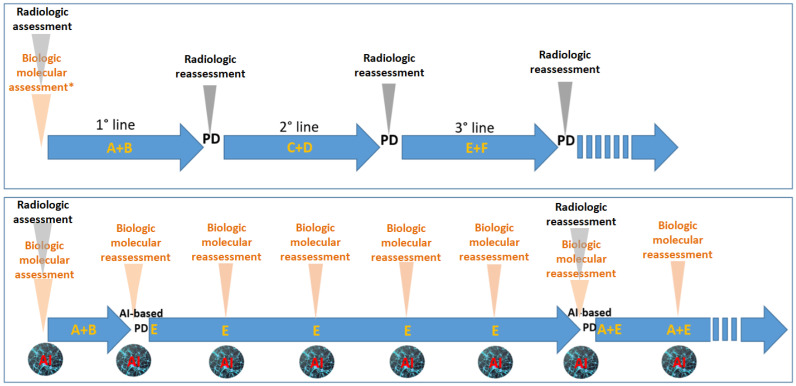
The figure highlights the differences between a standard oncologic treatment strategy (top panel) and a potential AI-driven innovative approach (bottom panel). In the conventional model, therapies are administered sequentially in predefined lines (e.g., first-line, second-line, etc.) based on non-cross-resistant combinations. These decisions are primarily guided by periodic radiological evaluations. Genomic profiling—typically performed on the primary tumor—plays a pivotal role in initial treatment planning (e.g., assessment of *RAS*/*BRAF* or MSI status in colorectal cancer), but subsequent treatment choices are predominantly informed by imaging. The bottom panel illustrates a theoretical AI-integrated strategy. Here, periodic genomic reassessments capture the dynamic evolution of the tumor—whether spontaneous or therapy-induced. In this model, AI-based interpretation of serial molecular data could detect the emergence of resistance to drug combination A+B, prompting the early initiation of drug E, even before radiological progression is apparent. Further AI-guided evaluations suggest continuation of E until a subsequent reassessment supports rechallenge with A in combination with E. This conceptual framework envisions a reduced reliance on imaging, favoring biologic/molecular assessments (including circulating tumor DNA [ctDNA] genomic data, digital pathology features, and proteomic profiles). Such an approach may better accommodate the inherent complexity and heterogeneity of cancer biology. A, B, C, D, E, F: different anticancer agents; AI: artificial intelligence; PD: progressive disease.

This early molecular insight allows for timely therapeutic adjustments and may also reveal co-occurring alterations that can be co-targeted in a synergistic manner—offering a strategy to overcome the limited efficacy often associated with single-agent therapies. However, capturing and interpreting the full spectrum of tumor heterogeneity through serial liquid biopsies presents a formidable analytical challenge. The volume of data generated by these longitudinal analyses increasingly exceeds our capacity for interpretation, creating a critical bottleneck in the translation of molecular complexity into actionable therapeutic strategies. At the intersection of oncology and AI, however, a transformative opportunity is emerging [25]. In fact, bioinformatics and AI are indispensable in this context, allowing for the extraction of actionable molecular signatures from complex and large multi-omic datasets—including genomic, transcriptomic, epigenomic, proteomic, and metabolomic layers. These integrative models will not only inform dynamic treatment selection but also support the rational design of multi-targeted therapeutic strategies aimed at circumventing resistance mechanisms. In such models, genomic data are continuously integrated into AI algorithms to determine whether therapy should be maintained, intensified, or modified over time (Figure 2).

Ultimately, the transformation of liquid biopsy from a descriptive to a predictive—and eventually prescriptive—clinical tool hinges on interdisciplinary collaboration among oncologists, molecular scientists, bioinformaticians, and data scientists. Longitudinal patient cohorts and adaptive clinical trials incorporating serial liquid biopsies will be essential for validating biomarkers of therapeutic efficacy and resistance [26], paving the way for a truly personalized oncology paradigm.

## 5. Not Only the Tumor, but Also Its Microenvironment: A Critical Determinant of Therapy Response

In this context, a key contributor to cancer complexity and therapeutic resistance is the TME, which plays a central role in promoting immune evasion, metabolic adaptation, and the formation of immunosuppressive niches [27]. The TME consists of a complex ecosystem of immune cells, fibroblasts, endothelial cells, extracellular matrix components, and soluble mediators that collectively shape tumor behavior and influence response to therapy.

Unlike tumor cells, the components of the TME are predominantly composed of genetically stable, non-malignant cells with more static biological features [28]. A limitation of liquid biopsy is its inability to directly characterize the TME [29]. While associations between tumor genomic alterations and specific TME profiles are plausible, they remain poorly defined and far from being systematically validated. Therefore, within a holistic view of cancer biology, a comprehensive analysis of tumor tissue remains indispensable.

Notably, the complexity of the TME is increasingly recognized as a key determinant of therapeutic outcome, reinforcing the value of integrated spatial and temporal profiling. Technologies such as multiplex immunohistochemistry, spatial transcriptomics, and single-cell RNA sequencing offer high-resolution characterization of the cellular and molecular interactions occurring within the tumor and its microenvironment [30,31]. These approaches complement liquid biopsy by providing spatial context and cellular specificity, which are essential for understanding the dynamic interplay between tumor cells and their surrounding niche.

Indeed, recent studies have shown that clonal diversity and cooperation among tumor subclones can modulate the immune landscape, promoting tumor progression and metastatic dissemination [32]. Spatial and temporal heterogeneity within the TME adds yet another layer of complexity, directly impacting the efficacy of immunotherapies and other targeted strategies [33]. The interaction between tumor cells and the TME is bidirectional: while the microenvironment influences tumor evolution, tumor-derived signals actively remodel the TME to support survival, proliferation, and immune escape.

The integration of AI into digital pathology and precision oncology is transforming modern cancer care, particularly by offering novel insights into the TME. In this context, AI enables the integration and interrogation of large-scale, multi-dimensional biological datasets, uncovering complex spatial and molecular patterns within the TME that would be difficult to discern through conventional analysis [34]. Deep learning models trained on multi-omic inputs can now predict protein structures, model molecular interactions, and anticipate tumor evolution under therapeutic pressure [35]. This predictive capability is already informing rational drug design, virtual screening, and the real-time generation of patient-specific therapeutic hypotheses [36,37].

AI-driven approaches applied to histopathology are advancing rapidly. Novel architectures, such as dual-path neural networks [38] and multi-instance learning models coupled with foundation models [39], enable the integration of morphological data from whole slide images (WSI) with spatially resolved transcriptomic and proteomic data. These models have been shown to accurately predict molecular subtypes of complex tumors, such as gliomas or virus-associated cancers [38,40,41]. Furthermore, computational techniques such as Histology-Inferred Protein Information (HIPI) now allow the inference of multiplexed protein expression directly from H&E-stained slides, enhancing both spatial and molecular resolution [42]. These innovations are redefining diagnostic paradigms, enabling granular classification of tumors based on morphology, genomics, and immune features. Technologies for in situ mutation detection and spatial genomics have improved the ability to identify subclonal niches, providing essential information for personalized therapeutic strategies [43,44,45].

In the previous paragraph, we discussed liquid biopsy as a valuable tool for monitoring tumor dynamics. However, a recognized limitation of this approach is its inability to directly characterize the TME. Emerging research is beginning to address this gap through novel analytical strategies. For instance, methylation profiling of ctDNA can reflect the cell-of-origin and tissue-specific epigenetic states, indirectly informing on TME composition, including stromal and immune cell interactions [46]. Similarly, fragmentomic analyses, examining the size distribution, fragmentation patterns, and end motifs of ctDNA, are being explored as surrogates of chromatin structure and cellular processes within the TME [47,48]. Additionally, circulating extracellular vesicles and exosomal proteins are gaining attention as promising biomarkers that may capture immune-related features of the TME, such as T-cell suppression [49,50]. These approaches collectively hold potential to expand the interpretive power of liquid biopsies beyond tumor-intrinsic genomic alterations.

## 6. Synthetic Lethality: Mechanistic Foundations, Computational Discovery, and Therapeutic Opportunities

Synthetic lethality (SL) describes a genetic interaction in which the simultaneous perturbation of two genes—neither of which is lethal on its own—results in cell death. In oncology, one of these perturbations is typically a tumor-specific lesion (germline or somatic), whereas the second is an intentionally induced inhibition of a seemingly unrelated pathway [51]. The therapeutic appeal is straightforward: if a cancer cell harbors mutation A, then pharmacologically inhibiting partner B will selectively kill the malignant clone while sparing normal tissue that retains wild-type A. This concept, first observed in Drosophila and then formalized in yeast genetics, has matured into a pillar of precision oncology, most notably exemplified by the clinical success of PARP inhibitors in *BRCA1*/*2*-defective malignancies [52,53]. Yet, BRCA–PARP is merely the visible tip of a far broader network of lethal genetic dependencies that remain to be fully charted.

Tumor cells accumulate mutations that rewire signaling, metabolic, and DNA-repair circuits. Although these alterations confer growth advantages, they also create hidden liabilities because the cell’s homeostatic buffers—redundant or compensatory pathways—must take up the slack created by the primary lesion. When a compensatory node is disabled, the fragile new equilibrium collapses and the cell undergoes mitotic catastrophe, ferroptosis, or other fatal outcomes. For instance, *BRCA1/2* loss cripples homologous recombination repair; PARP inhibition further blocks base-excision repair, producing unrepaired single-strand breaks that convert into lethal double-strand lesions during replication [54]. Other well-studied pairs include *PTEN* loss with PI3Kβ blockade [55], *IDH1/2* mutations with glutaminase inhibition [56], *ARID1A* deficiency with EZH2 or ATR inhibitors [57], and *SMARCA4* inactivation with BRD9 degraders [58]. Across these examples, the synthetic-lethal partner is rarely the most obvious downstream effector of the driver lesion; instead, it is often a parallel pathway that has silently assumed essential housekeeping duties.

Early SL discovery relied on intuition-driven, low-throughput experiments. Today, genome-wide CRISPR and shRNA dropout screens in isogenic cell lines or patient-derived organoids systematically map lethal pairs across thousands of mutational contexts. These datasets, combined with transcriptional, proteomic, phospho-signaling, and metabolomic layers, have revealed that SL interactions are highly context-specific: a dependency observed in *KRAS*-mutant pancreatic cells may not hold in *KRAS*-mutant colorectal cells because of lineage-restricted circuitry [59]. Therefore, comprehensive biological insight—spanning DNA-repair, cell-cycle checkpoints, chromatin architecture, epigenetic state, metabolic flux, and immune crosstalk—is indispensable for prioritizing actionable vulnerabilities.

The combinatorial space of potential gene–drug or gene–gene interactions is astronomically large, outstripping human cognitive capacity. AI is uniquely suited to navigate this space by integrating heterogeneous omics, chemical, and clinical outcome datasets. Graph convolutional networks can embed genes, proteins, metabolites, and drugs into a unified knowledge graph, enabling the algorithm to infer probable SL edges based on topological proximity, shared ontologies, and learned patterns of co-essentiality [60]. Variational autoencoders trained on CRISPR screen matrices can generate latent representations that highlight conditional lethalities under specific mutational or microenvironmental constraints. Moreover, transformer-based language models pre-trained on biomedical literature (e.g., PubMedGPT variants) extract mechanistic cues from millions of abstracts, suggesting testable SL pairs that have never been experimentally probed [61,62].

Coupling these AI pipelines with in silico virtual screening closes the loop between target discovery and drug identification. For a predicted partner gene lacking bespoke inhibitors, algorithms can interrogate >20,000 clinically approved or investigational molecules for binding motifs, off-target polypharmacology, and ADMET profiles, rapidly nominating repurposing candidates [63]. The iterative cycle proceeds as follows: (i) AI predicts an SL interaction; (ii) docking and molecular-dynamics simulations identify druggable pockets; (iii) repurposed compounds or de novo-designed ligands are prioritized; (iv) in vitro viability assays and single-cell transcriptomics validate selective lethality; (v) feedback refines the model. Such closed-loop learning has already accelerated the path from computational hypothesis to clinically actionable insight in less than a year for certain targets [64].

SL is evolving into a dynamic, AI-driven concept. Beyond static gene pairs such as *BRCA*–*PARP*, new approaches identify context-specific and stress-induced vulnerabilities using knowledge graphs and single-cell data. AI can forecast when tumor clones will develop dependencies that can be targeted before resistance emerges. Moreover, linking SL discovery to in silico drug screening allows rapid identification of repurposable compounds. These strategies can personalize SL targeting across tumor types and timepoints, turning cancer’s adaptability into an opportunity for proactive, precision-based interventions.

## 7. Drug Repurposing and Uncovering Hidden Pharmacological Opportunities

Repurposed drugs—such as statins, metformin, bisphosphonates, non-selective β-blockers, and certain antifungals—frequently exhibit anticancer effects that were initially unexplained. Emerging evidence now indicates that many of these effects arise from context-dependent synthetic lethality (SL). For example, statins inhibit HMG-CoA reductase, reducing mevalonate-pathway flux; tumors bearing p53 gain-of-function mutations or alterations in the mevalonate—YAP/TAZ axis become exquisitely sensitive because they rely on prenylation-dependent small GTPases for survival [65]. Metformin, long known to activate AMPK and impair mitochondrial respiration, can be synthetically lethal in LKB1-deficient or mitochondrial-DNA-mutant contexts, where energetic buffering is already compromised [66]. Crucially, the pre-existing clinical safety data for such agents lowers the translational barrier, facilitating rapid initiation of biomarker-driven trials.

However, as with targeted therapies, repurposed drugs should not be administered indiscriminately to unselected patient populations. Instead, their clinical use ought to be guided by specific molecular or metabolic features that predict therapeutic vulnerability. Administering these agents without biological stratification risks diluting their apparent clinical efficacy, as only a subset of patients may harbor the molecular dependencies required for response. This principle underscores the necessity of integrating biomarker-based patient selection even in the context of drug repurposing strategies. Beyond these examples, Table 2 summarizes selected repurposed drugs, their approved indications, reported anti-cancer activity, mechanisms of action, and possible rational selection of patients based on tumor biology.

SL extends beyond coding mutations. Epigenetic silencing of tumor suppressors (e.g., *MLH1* promoter methylation) creates dependencies exploitable by DNMT or HDAC inhibitors in combinatorial regimens [67]. Similarly, non-coding RNAs that fine-tune pathway redundancy can create Achilles’ heels: an oncogenic lncRNA that buffers DNA-damage tolerance might render the cell dependent on alternative repair routes, which can be blocked pharmacologically [68,69]. AI models trained on chromatin-accessibility and RNA-interactome maps are beginning to reveal these subtler layers of SL, which were invisible to gene-centric screens.

Despite its promise, clinical translation of SL faces hurdles. First, tumor heterogeneity means the biomarker lesion may not be ubiquitous across all metastatic sites, risking outgrowth of resistant clones. Second, many synthetic-lethal inhibitors target ubiquitous cellular processes (e.g., replication stress), raising toxicity concerns. AI can mitigate these issues by modelling clone-level heterogeneity from liquid-biopsy sequencing, predicting the likelihood of pre-existing escape variants, and simulating therapeutic windows across normal tissue transcriptomes. Integration of differential gene-essentiality data from CRISPR screens in normal organoids further informs safety profiles [70].

The future lies in the dynamic exploitation of SL. Serial ctDNA sampling combined with AI-driven evolutionary forecasting will detect emergent subclones and suggest the next-line SL partner before clinical progression. Master-protocol trials, embedding Bayesian adaptive randomization, can test multiple SL-guided combinations in parallel, continuously updating enrolment criteria based on accumulating molecular response data [71,72]. In silico twin models of each enrolled patient—fed by longitudinal multi-omics—will simulate various intervention sequences, nominating the regimen with the highest probability of durable response.

SL crystallizes the essence of precision oncology: turning cancer’s own wiring errors into selective liabilities. To fully unlock this therapeutic space, a comprehensive understanding of cellular biology under stress—including compensatory signaling, metabolic flux, and chromatin remodeling—is required. AI, with its capacity to integrate vast biological knowledge and uncover latent patterns, is the indispensable ally in this endeavor. By marrying AI-driven discovery with pragmatic drug repurposing and adaptive clinical designs, the field stands poised to translate the theoretical elegance of synthetic lethality into tangible, patient-centered benefit.

Thus, these recent advances reposition drug repurposing as a precision-guided strategy rather than “opportunistic” and empiric reuse. AI models now integrate tumor genomics, drug-target networks, and treatment evolution to predict when a repurposed drug (such as metformin or statins) can trigger SL in specific molecular contexts. Reinforcement learning can simulate effective combinations and dosing [73], while adaptive trial designs enable dynamic patient reassignment based on biomarkers. Public pharmacogenomic datasets combined with AI tools help uncover unexpected therapeutic matches, transforming repurposed agents into tailored treatments for biomarker-defined cancer subtypes.

## 8. Conceptual Framework Narrative: AI-Driven Oncology Model

**Figure 3:**
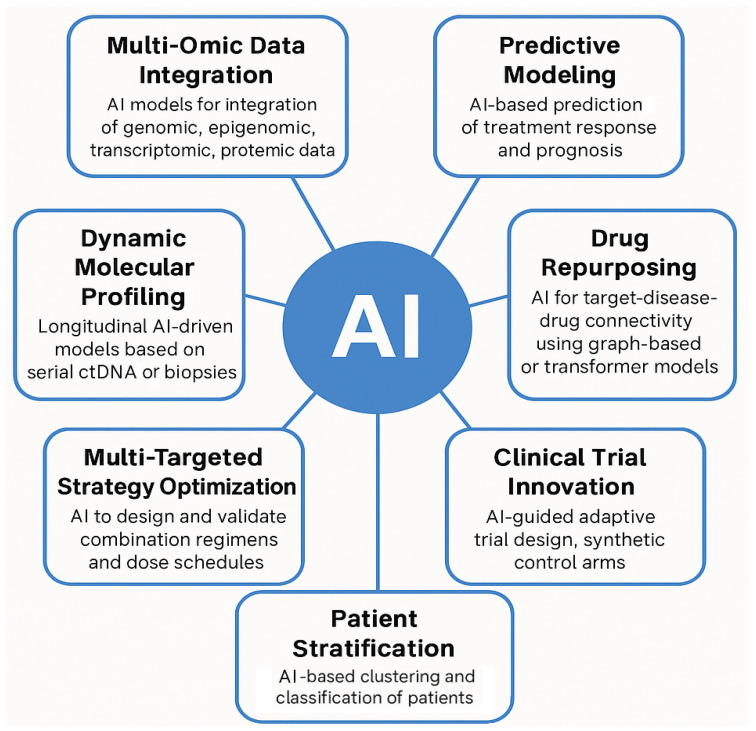
This figure proposes an integrated conceptual framework for how artificial intelligence (AI) can be deployed to confront the multidimensional complexity of cancer in the post-genomic era. The framework is organized into seven functional domains, each addressing a specific layer of oncological complexity (from molecular integration to therapeutic optimization and clinical innovation). Importantly, Multi-Omic Data Integration and Dynamic Molecular Profiling represent critical input layers of the model, serving as individualized and longitudinal data sources that feed the AI-driven architecture. These inputs enable downstream modules to perform predictive modeling, therapeutic reasoning (e.g., drug repurposing and multi-targeted combination design), patient stratification, and adaptive trial structuring. The distinction between input domains and actionable output domains reflects a conceptual workflow, where AI not only processes vast datasets but actively generates clinically actionable insights, reinforcing its role as a therapeutic enabler rather than a passive analytical tool.

In the post-genomic oncology paradigm, AI serves as a central integrative engine that unifies patient-specific molecular inputs and dynamic profiling to drive predictive modeling, therapeutic repurposing, combination strategy optimization, patient stratification, and novel clinical trial design. At its core, this framework begins with multi-omic data integration, where AI synthesizes genomics, epigenomics, transcriptomics, proteomics, and metabolomics data to delineate tumor heterogeneity and uncover latent biomarkers [74]. Complementing this is dynamic molecular profiling, leveraging longitudinal samples such as ctDNA or serial biopsies, enabling deep learning models to capture temporal tumor evolution and emerging resistance mechanisms. Next, predictive modeling uses these fused datasets to forecast treatment response and prognosis at the individual level, accommodating inter-patient variability. In parallel, AI-driven drug repurposing applies graph-based, transformer, or network-mining methods to identify novel therapeutic uses for existing agents, expediting clinical translation [75]. The framework further advances with multi-targeted strategy optimization, where reinforcement learning and combination-design algorithms propose synergistic regimens and dose schedules tailored to multi-pathway dependencies. By integrating this information, AI can classify subpopulations with distinct phenotypic and therapeutic response profiles. Finally, AI-guided clinical trial innovation offers adaptive designs and synthetic control arms that enhance trial efficiency, which is especially beneficial for rare molecular subtypes. This conceptual model provides an organizational structure, linking each AI application domain to specific aspects of cancer complexity. It positions AI not merely as a prognostic or predictive tool but as a therapeutic enabler, fostering precision interventions informed by layered data inputs (Figure 3).

## 9. Barriers and Limitations in the Clinical Implementation of AI in Oncology

While the integration of AI into oncology holds transformative potential, a balanced and realistic perspective must account for the substantial practical challenges and systemic limitations that hinder clinical implementation. First, economic constraints represent a major barrier. The costs associated with serial NGS, multi-omics profiling, and high-resolution imaging (necessary for feeding AI models with robust input) remain prohibitive for many healthcare systems. This raises concerns regarding equitable access to AI-guided precision oncology, particularly in under-resourced or publicly funded contexts where reimbursement pathways for such technologies are unclear or absent. Second, regulatory hurdles remain significant. The complexity, adaptiveness, and opacity of many AI algorithms (particularly those employing deep learning) pose difficulties for standard validation, auditing, and approval by regulatory agencies such as the EMA or FDA. Current frameworks are better suited to static diagnostics or single-gene tests than to continuously learning, multifactorial models that evolve as new data are incorporated. Third, data infrastructure and standardization issues further complicate deployment. AI requires large, harmonized datasets that integrate molecular, clinical, and radiological data; yet, in practice, such datasets are fragmented across institutions, lack standardized formats, and often suffer from batch effects or inconsistent bioinformatics pipelines. Without uniform wet-lab protocols and interoperable data architectures, reproducibility and model generalizability remain compromised.

In addition to these operational challenges, it is crucial to acknowledge the intrinsic limitations and risks of AI itself. Despite its impressive analytical capabilities, AI is not a panacea. Many deep learning systems operate as “black boxes,” offering high accuracy without interpretability, an issue that undermines clinician trust and poses ethical and legal dilemmas in medical decision-making. Furthermore, AI models trained on retrospective, often Western-centric datasets risk perpetuating or amplifying biases, particularly in patient populations that are underrepresented in training cohorts. This can lead to suboptimal or even harmful recommendations when deployed broadly. Finally, the translation of AI-derived therapeutic hypotheses into clinical benefit requires prospective validation in controlled clinical trials, a step that remains underdeveloped in current literature and practice. Without rigorous validation, there is a danger of overfitting to past data rather than truly predicting future outcomes. Thus, while the promise of AI in oncology is considerable, realizing its full potential requires careful navigation of these technical, logistical, and ethical challenges through interdisciplinary collaboration, regulatory innovation, and continued investment in infrastructure and clinical validation.

## 10. Conclusions: Towards Continuously Responsive Oncology

The reductionist model of single-target, single-agent therapies must give way to rational and dynamic multi-targeted strategies that account for the adaptive and evolving nature of cancer. Beyond serving as computational accelerants, AI platforms may evolve into true hypothesis-generating engines, capable of identifying emergent vulnerabilities and designing adaptive, multi-target therapeutic approaches. This is especially relevant not only for identifying novel actionable direct targets but also in complex contexts such as synthetic lethality, where targeting compensatory pathways can induce tumor collapse.

To realize this potential, the integration of real-time liquid biopsy with serial genomic profiling and AI-supported decision-making may enable “continuously responsive oncology,” wherein treatment regimens are dynamically adapted based on longitudinal tumor evolution. Predictive models may soon forecast not only resistance mutations but also their timing and likelihood, allowing preemptive therapeutic reprogramming.

Future clinical trials may increasingly adopt adaptive designs, comparing conventional line-based therapies with AI-guided approaches in real time. Within such frameworks, therapeutic decisions are continuously optimized.

Ultimately, a systems biology approach enhanced by AI may enable a paradigm shift from reactive treatment to anticipatory control of cancer, improving long-term outcomes and transforming therapeutic intent from disease management to durable remission or cure.

## Figures and Tables

**Table 1:** Improved clinical responses with combination targeted therapies across selected solid tumors.

Cancer Type	Monotherapy	Outcome with Monotherapy	Combination Therapy	Outcome with Combination Therapy
Metastatic colorectal cancer (KRAS G12C+)	Sotorasib (KRAS G12C inhibitor)	ORR: 0%	Sotorasib + Panitumumab (anti-EGFR mAb)	ORR: 26.4%
HER2-positive breast cancer (neoadjuvant)	Trastuzumab (anti-HER2 mAb)	pCR: 29.5%	Trastuzumab + Lapatinib (HER2 TKI)	pCR: 51.3%
HR+/HER2+ metastatic breast cancer	Trastuzumab + endocrine therapy	ORR: 13.7%	Trastuzumab + Lapatinib + Aromatase inhibitor	ORR: 31.7%
BRAF V600-mutant metastatic melanoma	Vemurafenib (BRAF inhibitor)	ORR: 45%	Vemurafenib + Cobimetinib (MEK inhibitor)	ORR: 68%

ORR, objective response rate; pCR, pathologic complete response; mAb, monoclonal antibody; TKI, tyrosine kinase inhibitor; HR+, hormone receptor-positive.

**Table 2:** Selected non-oncologic drugs with potential for repurposing in cancer therapy.

Repurposed Drug	Approved Indication	Cancer Types Showing Response	Known Anti-Tumor Mechanism	Rational Selection of Cancer Patients
Metformin	Type II Diabetes	Breast, prostate, pancreatic, NSCLC	AMPK activation; inhibition of mitochondrial complex I; metabolic stress	Tumors with LKB1 deficiency or mtDNA mutations
Disulfiram	Alcohol dependence (aversion therapy)	Breast, glioblastoma, prostate, melanoma	Inhibition of ALDH; ROS accumulation; proteasome inhibition via Cu^2+^-complex	Tumors with high ALDH expression or low antioxidant defense
Propranolol	Hypertension, arrhythmias, migraine prophylaxis	Angiosarcoma, breast cancer, melanoma	Non-selective β-blockade; anti-angiogenic; suppression of β-adrenergic signaling	Tumors expressing β-adrenergic receptors; highly vascular tumors
Itraconazole	Fungal infections	Basal cell carcinoma, prostate, NSCLC	Hedgehog pathway inhibition; anti-angiogenic; mTOR and P-gp inhibition	Tumors with Hedgehog pathway activation; high angiogenic profile
Chloroquine/ Hydroxychloroquine	Malaria, autoimmune diseases	Glioblastoma, pancreatic, breast	Autophagy inhibition; lysosomal destabilization; immune modulation	RAS-mutant, hypoxic tumors, or autophagy-addicted cancers
Statins (e.g., Simvastatin)	Hypercholesterolemia	Breast, ovarian, colorectal, prostate	Inhibition of HMG-CoA reductase; disruption of prenylation (e.g., RAS); apoptosis induction	Tumors with RAS activation, mevalonate pathway dependency
Valproic Acid (VPA)	Epilepsy, bipolar disorder	Glioblastoma, breast, prostate, myeloid malignancies	HDAC inhibition; chromatin remodeling; re-expression of silenced tumor suppressor genes	Tumors with epigenetic silencing (e.g., low histone acetylation, hypermethylation); tumors with low expression of immune-regulatory genes

ALDH: Aldehyde dehydrogenase; AMPK: AMP-activated protein kinase; HDAC: Histone deacetylase; LKB1: Liver kinase B1; mTOR: Mechanistic target of rapamycin; mtDNA: Mitochondrial DNA (mutations may impair respiration and increase sensitivity to metabolic inhibitors); NSCLC: Non-small cell lung cancer; P-gp: P-glycoprotein; RAS: Family of small GTPases including HRAS, KRAS, and NRAS; ROS: Reactive oxygen species.

## Data Availability

No new data were generated or analyzed in this study.

## Abbreviations

The following abbreviations are used in this manuscript:

ADMET	Absorption, Distribution, Metabolism, Excretion, and Toxicity
AKT	AKT Serine/Threonine Kinase
ALK	Anaplastic Lymphoma Kinase
AMPK	AMP-Activated Protein Kinase
ARID1A	AT-Rich Interaction Domain 1A
ATR	Ataxia Telangiectasia and Rad3-Related Protein
BCR-ABL1	Breakpoint Cluster Region–Abelson Murine Leukemia Viral Oncogene Homolog 1
BRAF	B-Raf Proto-Oncogene, Serine/Threonine Kinase
BRCA1/2	Breast Cancer 1/2 Genes
CML	Chronic Myeloid Leukemia
CRISPR	Clustered Regularly Interspaced Short Palindromic Repeats
ctDNA	Circulating Tumor DNA
DNMT	DNA Methyltransferase
DTP	Drug-Tolerant Persister
EGFR	Epidermal Growth Factor Receptor
EZH2	Enhancer of Zeste Homolog 2
GTPases	Guanosine Triphosphatases
HDAC	Histone Deacetylase
HER2	Human Epidermal Growth Factor Receptor 2
HIPI	Histology-Inferred Protein Information
HMG-CoA	3-Hydroxy-3-Methylglutaryl-Coenzyme A
HR	Hormone Receptor
IDH1/2	Isocitrate Dehydrogenase 1/2
ITH	Intratumoral Heterogeneity
KRAS	Kirsten Rat Sarcoma Viral Oncogene Homolog
lncRNA	Long Non-Coding RNA
LKB1	Liver Kinase B1
mAb	Monoclonal Antibody
MEK	Mitogen-Activated Protein Kinase Kinase (MAP2K)
MLH1	MutL Homolog 1
NGS	Next-Generation Sequencing
NRAS	Neuroblastoma RAS Viral Oncogene Homolog
NSCLC	Non-Small Cell Lung Cancer
NTRK	Neurotrophic Tyrosine Receptor Kinase
ORR	Objective Response Rate
PARP	Poly (ADP-ribose) Polymerase
pCR	Pathologic Complete Response
PD	Progressive Disease
PI3K	Phosphoinositide 3-Kinase
PTEN	Phosphatase and Tensin Homolog
shRNA	Short Hairpin RNA
SL	Synthetic Lethality
SMARCA4	SWI/SNF Related, Matrix Associated, Actin Dependent Regulator of Chromatin Subfamily A Member 4
TME	Tumor Microenvironment
TKI	Tyrosine Kinase Inhibitor
WES	Whole-Exome Sequencing
YAP/TAZ axis	Yes-Associated Protein/Transcriptional Coactivator with PDZ-Binding Motif Axis
